# Diversity of Global Rice Markets and the Science Required for Consumer-Targeted Rice Breeding

**DOI:** 10.1371/journal.pone.0085106

**Published:** 2014-01-14

**Authors:** Mariafe Calingacion, Alice Laborte, Andrew Nelson, Adoracion Resurreccion, Jeanaflor Crystal Concepcion, Venea Dara Daygon, Roland Mumm, Russell Reinke, Sharifa Dipti, Priscila Zaczuk Bassinello, John Manful, Sakhan Sophany, Karla Cordero Lara, Jinsong Bao, Lihong Xie, Katerine Loaiza, Ahmad El-hissewy, Joseph Gayin, Neerja Sharma, Sivakami Rajeswari, Swaminathan Manonmani, N. Shobha Rani, Suneetha Kota, Siti Dewi Indrasari, Fatemeh Habibi, Maryam Hosseini, Fatemeh Tavasoli, Keitaro Suzuki, Takayuki Umemoto, Chanthkone Boualaphanh, Huei Hong Lee, Yiu Pang Hung, Asfaliza Ramli, Pa Pa Aung, Rauf Ahmad, Javed Iqbal Wattoo, Evelyn Bandonill, Marissa Romero, Carla Moita Brites, Roshni Hafeel, Huu-Sheng Lur, Kunya Cheaupun, Supanee Jongdee, Pedro Blanco, Rolfe Bryant, Nguyen Thi Lang, Robert D. Hall, Melissa Fitzgerald

**Affiliations:** 1 International Rice Research Institute, Los Baños, Laguna, Philippines; 2 Laboratory of Plant Physiology, Wageningen University, Wageningen, The Netherlands; 3 International Network for Quality Rice; 4 Plant Research International, Wageningen, The Netherlands; 5 Centre for BioSystems Genomics, Wageningen, The Netherlands; 6 Yanco Agricultural Institute, NSW Department of Industry and Investment, PMB, Yanco, New South Wales, Australia; 7 Grain Quality and Nutrition Division, Bangladesh Rice Research Institute (BRRI), Gazipur, Bangladesh; 8 EMBRAPA Rice and Beans, Santo Antonio de Goiás, GO, Brazil; 9 Africa Rice Center (AfricaRice), Cotonou, Republic of Benin; 10 Cambodian Agricultural Research and Development Institute, Phnom Penh, Cambodia; 11 Mejoramiento Genetico de Arroz INIA CRI Quilamapu, Vicente Mendez, Chile; 12 Institute of Nuclear Agricultural Sciences, Zhejiang University, Hua Jiachi Campus, Hangzhou, China; 13 China National Rice Research Institute, Hangzhou, China; 14 Laboratorio de Calidad FLAR-CIAT, CIAT, Cali-Palmira, Colombia; 15 Rice Research Section, Field Crops Research Institute, A.R.C., Rice Research & Training Center, Sakha, Kafr El-Shiekh, Egypt; 16 CSIR-Food Research Institute, Accra, Ghana; 17 Rice Section, Department of Plant Breeding and Genetics, Punjab Agricultural University Ludhiana, Ludhiana, India; 18 Department of Rice, Centre for Plant Breeding and Genetics, Tamil Nadu Agricultural University Coimbatore, Tamil Nadu, India; 19 Crop Improvement Section, Directorate of Rice Research, Rajendra Nagar, Hyderabad, AP, India; 20 Indonesian Center for Rice Research (ICRR) BB Padi, Sukamandi, Subang Jawa Barat, Indonesia; 21 Rice Research Institute of Iran (RRII), Rasht, I.R. Iran; 22 NARO Institute of Crop Science, 2-1-18 Kannondai, Tsukuba, Ibaraki, Japan; 23 NARO Hokkaido Agricultural Research Centre, Toyohira, Sapporo, Hokkaido, Japan; 24 Rice and Cash Crop Research Institute, NAFRI, Vientiane, Lao PDR; 25 Faculty of Agriculture and Food Science, Universiti Putra Malaysia, Bintulu Sarawak, Malaysia; 26 Pusat Penyelidikan Padi dan Tanaman Industri, MARDI Seberang Perai Beg Berkunci, Seberang Perai Pulau Penang, Malaysia; 27 Plant Biotechnology Center, Myanmar Agriculture Service, Ministry of Agriculture and Irrigation, Yangon, Myanmar; 28 Rice Programme, National Agricultural Research Centre, Islamabad, Pakistan; 29 National Institute for Biotechnology and Genetic Engineering, Faislabad, Pakistan; 30 Rice Chemistry and Food Science Division, Philippine Rice Research Institute, Maligaya, Science City of Muñoz, Nueva Ecija, Philippines; 31 Instituto Nacional de Investigacao Agraria e Veterinaria, Quinta do Marques, Oeiras, Portugal; 32 Rice Research Station, Department of Agriculture, Ambalantota, Sri Lanka; 33 Department of Agronomy, National Taiwan University, Taiwan; 34 Pathumthani Rice Research Centre, Bureau of Rice Research and Development, Thailand; 35 Khon Kaen Rice Research Center, Khon Kaen, Thailand; 36 Rice Research Program, National Agricultural Research Institute, INIA Treinta y Tres, Treinta y Tres, CP, Uruguay; 37 USDA-ARS, Dale Bumpers National Rice Research Center, Stuttgart, Arkansas, United States of America; 38 Genetic & Plant Breeding Division, Cuulong Delta Rice Research Inst., Can Tho, Viet Nam; National Rice Research Center, United States of Ameirca

## Abstract

With the ever-increasing global demand for high quality rice in both local production regions and with Western consumers, we have a strong desire to understand better the importance of the different traits that make up the quality of the rice grain and obtain a full picture of rice quality demographics. Rice is by no means a ‘one size fits all’ crop. Regional preferences are not only striking, they drive the market and hence are of major economic importance in any rice breeding / improvement strategy. In this analysis, we have engaged local experts across the world to perform a full assessment of all the major rice quality trait characteristics and importantly, to determine how these are combined in the most preferred varieties for each of their regions. Physical as well as biochemical characteristics have been monitored and this has resulted in the identification of no less than 18 quality trait combinations. This complexity immediately reveals the extent of the specificity of consumer preference. Nevertheless, further assessment of these combinations at the variety level reveals that several groups still comprise varieties which consumers can readily identify as being different. This emphasises the shortcomings in the current tools we have available to assess rice quality and raises the issue of how we might correct for this in the future. Only with additional tools and research will we be able to define directed strategies for rice breeding which are able to combine important agronomic features with the demands of local consumers for specific quality attributes and hence, design new, improved crop varieties which will be awarded success in the global market.

## Introduction

Economic growth in Asia over the past thirty years has increased incomes and lifted millions out of poverty [1]. Globally, confidence is growing that the first Millennium Development Goal (to cut extreme hunger and poverty by half) will be met, and that economic growth will continue, and lift millions more from poverty [2]. It is projected that by 2050 Asia will account for half of the world’s economic output [3], and be home to the largest proportion of middle classes, who will increasingly demand higher quality food, including higher quality rice [3]–[6]. However, coupled with economic growth in Asia, population pressure is increasing, agricultural land is being lost to urbanisation, and there is significant loss of workers from farms as the younger generation moves to cities [2]. Furthermore, climate change is predicted to have a severe impact on agricultural production in Asian countries [7].

Rice consumption per capita in many Asian countries is decreasing steadily due to changing dietary habits as a result of western influence, such as increased intake of dairy, meat and fast foods, and economic development [8]. Despite this decline, current projections of population growth indicate that an additional 8 million tonnes of rice must be produced each year in Asia [9]. Furthermore, western countries have started eating rice regularly [10], and continued growth of Asian communities within western countries has increased the market size for rice as well as creating awareness and appreciation of rice-based meals [11]. Taken together, for a developing Asia these factors all indicate that resource use efficiency must increase significantly in future rice production, and must meet the dual requirements of increasing production with fewer resources, while meeting the market demands of increasingly discerning consumers.

Many countries still grow traditional varieties of rice such as Khao Dawk Mali 105, selected in 1958 in Thailand [12], or popular, long-standing, improved varieties such as IR64 released in 1985 in the Philippines [12] and Swarna released in 1979 in India. This is despite significant national investment in rice improvement programs and continual release of new, high-yielding varieties. Adoption of these improved varieties by rice farmers is conditional upon consumer acceptance of the sensory and cooking properties of the grain [13]. People prefer a specific type of rice for a number of reasons. A survey conducted in Cambodia revealed that different quality traits were prioritised by different actors in the rice value chain. These traits included grain shape and appearance, aroma, texture and lack of chalk (ACIAR CSE-2009-005 Rice Market Survey). Consumers are readily able to determine if the sensory properties of a new variety are acceptable. However, when a new variety is not acceptable, it is difficult for consumers to describe why, and this makes it difficult for rice research programs to conduct the type of research that will deliver relevant selection tools to breeding programs.

Most of the studies conducted on rice consumer preferences have focused on the requirements of a particular country [14]–[21], but the rice market is now global, and there is a need for a more holistic perspective on consumer preferences relating to rice grain quality and its geographic variability. This will allow for a more targeted approach towards developing and disseminating new rice varieties which have an increased probability of adoption and acceptance.

The establishment of the International Network for Quality Rice (INQR), made it possible to conduct a survey of consumer preferences for rice quality in different rice-consuming regions. The INQR serves as the platform for all experts working on rice grain quality to: exchange information; establish new and standardised protocols for measuring different quality parameters; and identify new traits of physical, sensory and nutritional quality (https://inqr.irri.org). Through the INQR, experts in 23 countries participated in the survey reported here.

In the present paper we describe an extensive analysis of regional preferences for the main features of rice quality that are routinely measured in quality evaluation programs – length and shape of the grain, amylose content, gelatinisation temperature, gel consistency and aroma. Our objectives are to understand the specific market requirements for rice in each country in Asia as close to the provincial level as possible, and in some of Asia’s export markets, and to identify gaps that investment and research must fill to increase the ability of rice breeders to develop high-yielding, climate-ready varieties with the quality traits required by the consumers in their local markets.

## Methods

### Data collection and analysis

A survey was conducted among the members of the INQR. They were asked to identify the three most popular varieties of rice from their respective countries, and where possible disaggregated to the provincial, regional or state level (Table S1). Information was supplied for the commonly measured quality traits of grain length and shape, amylose content, gel consistency, gelatinisation temperature and aroma. For countries unable to measure all traits, samples of the chosen varieties were sent to the International Rice Research Institute (IRRI) for phenotyping of those traits. The consumer preferences for grain quality traits presented here refer to the characteristics of the three most popular varieties identified per region (country, state or province). Although variations in preferences exist within a region, only the dominant preferences are captured here.

Data for each quality trait was mapped in units based on administrative level (e.g., whole country, state, province) using boundaries denoted in the Global Administrative Areas (GADM) database (www.gadm.org). There are a total of 92 spatial units for which we have data on preference for at least two grain quality traits (Figure S1).

Data from the survey were classified as previously defined [22], [23]. The classes of grain length of milled rice in Asian rice improvement programs are defined as short (<5.5 mm), medium (5.51–6.6 mm), long (6.61–7.5 mm) and extra-long (>7.51 mm) [22]. These definitions differ from those described in the CODEX standard (198-1995) and by the European Commission [24], both of which define long grains as being 6 mm or more. For the purposes of this paper, which focuses on Asian rice, we have retained the classes for grain dimensions defined by Khush et al. (1979), and used the classifications for all other traits that were described by Juliano et al. (1985). Grain shape (length/width) was classified as bold (<2), medium (2 – 3) or slender (>3). Amylose content was classified as waxy (∼ 0%), low (2 – 19%), intermediate (20–25%) or high (>25%). Categories for gel consistency were soft (>60 mm), intermediate (40–60 mm) or hard (<40 mm). Gelatinisation temperature was classified as low (<70°C), intermediate (70–74°C) or high (>74°C).

The potential market share of each country was estimated based on FAO data on rice consumption per capita over a period of 20 years (Table S2) from 1990–2009 (the most recent available year: [25]). The countries in the INQR dataset account for over 90% of the global rice consumption, indicating that the data cover a substantial proportion of the global rice market.

### Length and shape of the grain

At IRRI, the length and width of grains were measured using a Cervitec Grain Inspector 1625 (FOSS Analytical, Hoganas, Sweden). For grain lengths reported from elsewhere, length and width were measured using calipers or a calibrated scanner.

### Amylose content

#### Colourimetric method

Amylose content was measured using the standard iodine colourimetric method ISO 6647-2-2011 [26]. Briefly, ethanol (1 mL, 95%) and 1 M sodium hydroxide (9 mL) were added to rice flour (100 mg), and this was heated in a boiling water bath until gelatinisation of the starch occurred. After cooling, 1 M acetic acid (1 mL) and iodine solution (2 mL) were added and the volume was made up to 100 mL with Millipore water. The iodine solution was prepared by dissolving 0.2 g iodine and 2.0 g potassium iodide in 100 mL Millipore water. Absorbance of the solution was measured using an Auto Analyser 3 (Bran+Luebbe, Norderstedt, Germany) at 600 nm. Amylose content was quantified from a standard curve generated from absorbance values of 4 well-known standard rice varieties (IR65, IR24, IR64 and IR8).

#### Size exclusion chromatography

Rice samples with low, intermediate and high amylose were selected and analysed using SEC exactly as previously described [27]. Rice flour (50 mg) was gelatinised, then debranched with isoamylase (Pseudomonas, Megazyme, Wicklow, Ireland) at 50°C for 2h, with regular agitation. An aliquot of each debranched solution (40µL) was analysed using SEC (Waters, Alliance 2695,Waters,Milford, USA) equipped with an Ultrahydrogel 250 column (Waters).

#### Genotyping the Waxy gene

Genomic DNA was extracted from rice leaves of the low amylose varieties from Cambodia, Thailand, Australian, and Japan using the method described previously [28]. Leaf samples were frozen in liquid nitrogen and ground into fine powder prior to addition of extraction buffer. PCR amplification was performed using a G-Storm Thermal Cycler (model GS1, Gene Technologies Ltd, Essex, UK) in a 20-µL reaction volume containing forward and reverse primers (Table 2), designed with Primer 3 software [29], and components from KAPA HiFi HotStart PCR Reagent Kit (KAPA Biosystems, Boston, Massachusetts, USA). Twenty microliters of the PCR product were electrophoresed through a 1.2% agarose gel, stained with SybrSafe nucleic acid stain (Invitrogen, Carlsbad, CA, USA), and visualized using a non-ultraviolet transilluminator (Dark Reader DR195M, Clare Chemicals, Dolores, CO, USA). PCR fragments from agarose gels were purified using the QIAquick Gel Extraction Kit (Qiagen, Hilden, Germany) according to the manufacturer’s instruction. Sequencing of the PCR products was done by Macrogen Inc., Seoul, South Korea. Nucleotide sequences were retrieved and aligned using BIOEDIT software [30] with the Nipponbare rice *Wx* gene sequence from GRAMENE [31] as the reference.

### Gelatinisation temperature

Gelatinisation temperature was measured using a DSC Q100 instrument (TA Instrument, New Castle, DE, USA) [32]. Rice flour (4 mg) was mixed with Millipore water (8 mg) in hermetic aluminium pans which were then sealed. The pans were heated under pressure from 25 to 120°C at 10°C min^−1^. Some countries reported gelatinisation temperature as alkali spreading values, which were determined as previously described [33], and values ascribed to high, intermediate or low by the correlations reported previously [32].

### Gel consistency

Gel consistency was determined as previously described [34]. Rice flour (100 mg) was mixed with ethyl alcohol (0.2 mL) containing 0.025% thymol blue and 0.2 M potassium hydroxide (2 mL) and heated in a boiling water bath for 8 min. After heating, the sample tubes were allowed to cool in an ice-water bath and immediately laid horizontally on the table. Gel consistency was measured by the length of the cold rice paste in the culture tube held horizontally for one hour. Hard, medium and soft gel standards, IR48, PSBRC9 and IR42, respectively, were included in every set.

### Aroma

The current definition of aromatic rice is the presence of the volatile compound 2-acetyl-1-pyrroline (2AP). This was quantified at IRRI using gas chromatography (Agilent 6890N, Santa Clara, CA, USA) equipped with a flame ionisation detector [35]. For those rice samples not measured at IRRI, aroma was determined by smelling and tasting cooked grains.

### Volatile analysis of aromatic rice by gas chromatography- mass spectrometry (GCMS)

Volatile compounds in the aromatic rice from Iran, Pakistan, India and the Greater Mekong Sub-region (GMS) were analysed. Headspace volatile compounds of selected aromatic rice were collected by solid phase microextraction using a 65-mm polydimethylsiloxane-divinylbenzene fibre (Supelco, Bellefonte, USA) and analysed using GC-MS (GC 8000, Fisons Instruments, Cheshire, UK) [36]. GCMS raw data were processed using MetAlign [37] to extract and align the mass signals, and MSClust [38] to remove signal redundancy per metabolite and reconstruct mass spectra. The PCA plot was constructed using SIMCA-P 12.0 (Umetrics AB, Umea°, Sweden).

## Results and Discussion

Without an understanding of consumer preference for rice grain quality, wide adoption of any newly developed rice variety is not guaranteed. Hence, identifying the grain traits that govern acceptance is important to guide a successful breeding program. Quality attributes of the most popular rice varieties consumed in the countries and provinces of Asia, as well as for some of the rice-growing countries in other continents have been collected.

Currently, rice grain quality is classified in terms of its physical, cooking and sensory characteristics. The physical appearance of the grain defines its price in the market, whereas the cooking and sensory properties determine the reputation of the variety [39]. Grain appearance, the first thing a consumer sees, is defined by length, width and shape (ratio of length and width); and chalk, which consumers dislike [40] unless the variety is targeted towards use in paella or risotto. The physical traits of the grain are immediately recognisable even to an untrained eye. Cooking quality is a measure of the time and fuel required to cook the rice, and this is indirectly measured by gelatinisation temperature [32]. Sensory properties are influenced by amylose content [41], gel consistency [34], [42] and also gelatinisation temperature [43].

### Length and shape of the grain

**Figure 1:**
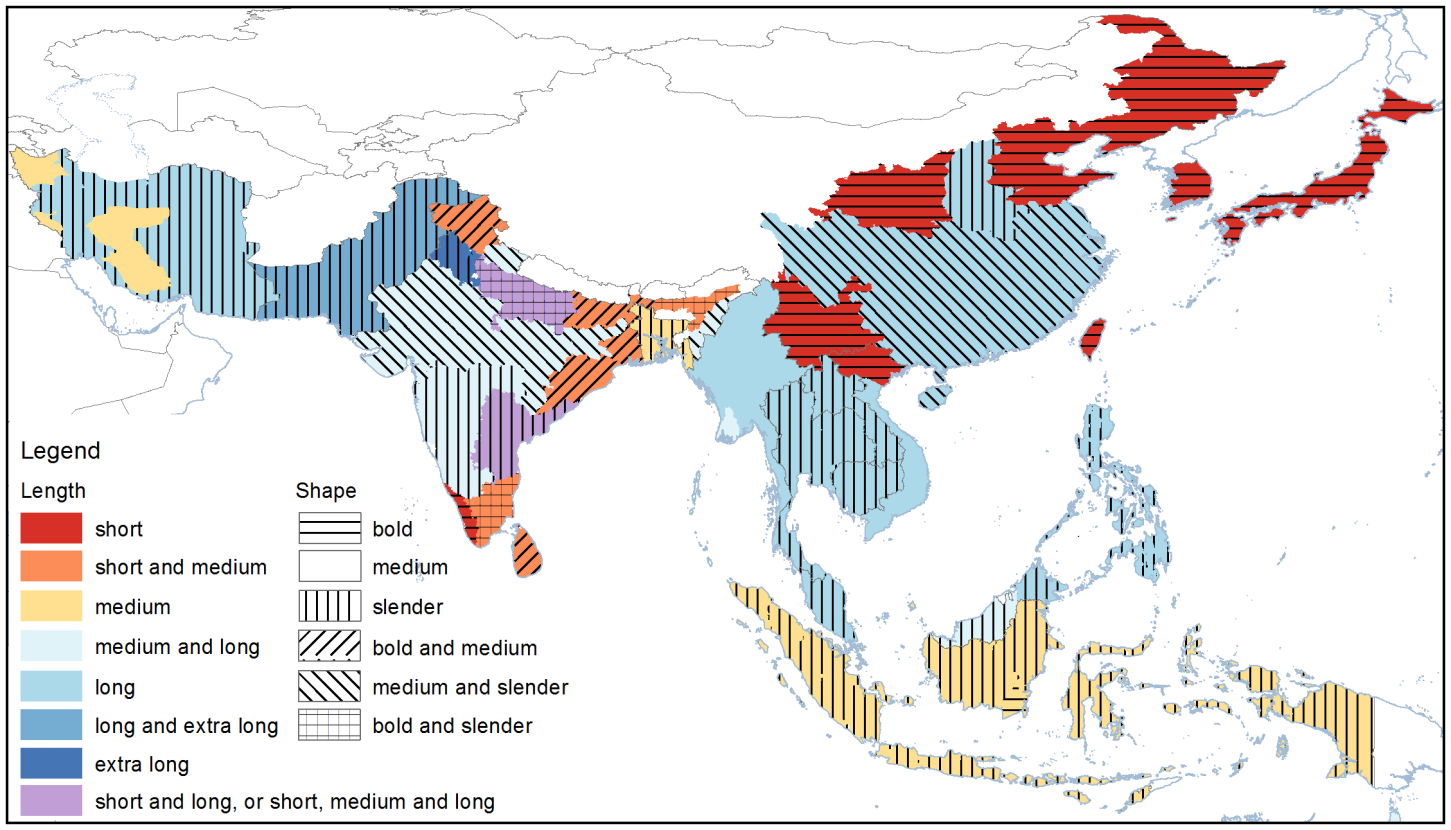
Regional variation in rice length and shape (length/width) of the three most popular varieties in the countries, states, and provinces of Asia. In some regions, more than one type of grains lengths and shapes are preferred. Colours represent length, and lines represent the shape. Additional information for other regions can be found in Table 2. Data were obtained from INQR representatives from each region.

The defined classes of grain length differ between Asian and European standards [22], [24], but for the purposes of this work, the Asian classification of length, as defined by Khush, et al. (1979) was used. Rice consumers in parts of South East Asia - Thailand, Lao PDR, Cambodia, Malaysia, and Philippines prefer long and slender grains (Figure 1). Consumers in Indonesia and Bangladesh prefer grains that are medium in length and slender. In North Asia, the Japanese, Taiwanese and South Koreans eat short and bold rice grains, whereas in several states of India, and Sri Lanka, both short and medium grains are popular. In most of Pakistan and the Indian states of Punjab and Haryana, the extra long grains characteristic of basmati rice are popular, and long grains are also popular in most of Iran and parts of Pakistan (Figure 1).

Rice consumers in the large countries of China, and India have regional differences in preferences for grain length and shape. Consumers in China prefer rice grains that are either short and bold, or are long in size but with the grain shape ranging from medium to slender depending on province. Indian rice preferences, on the other hand, are composed of an even more varied set of grain types- from short to medium, long and extra-long in length with shapes ranging from bold to slender (Figure 1).

Understanding the genetic basis of length should assist in harmonising the definitions of grain length between the European and Asian standards. Seven Quantitative Trait Loci (QTLs) for grain length have been reported and three for grain width [44]. Biallelic variation at the *GS3* locus appears to exert the most control over grain length [45]. All the short and medium grains genotyped carry the C-allele and all the long and extra-long grains carry the A-allele [46], [47]. However the association studies done for the gene *GS3* were all on paddy rice, and not on polished rice [45]–[47], so the range of white grain length of those carrying the C and A alleles is not yet known, and this information would enable the differences between the standards to be resolved.

**Figure 2:**
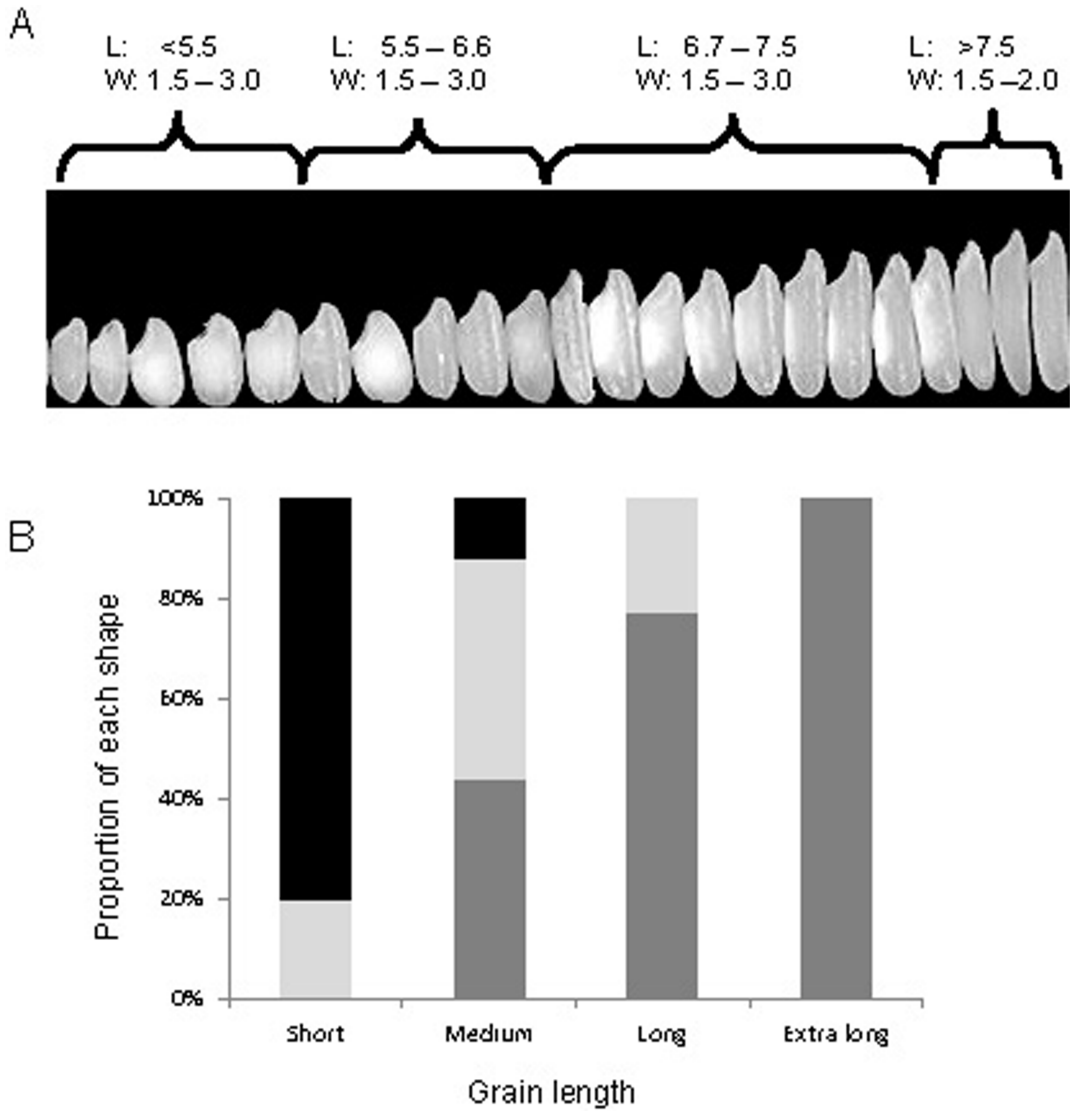
A: Grains ordered from shortest to longest, showing different widths (mm) in each class except the extra-long class. B: Histogram showing the proportion of bold (black), medium (light grey) and slender (dark grey) grain shapes within each length class for all the popular rices discussed in the present paper.

Figure 2A shows four clear groups for grain length. Koshihikari from Japan and Pandan Wangi from Indonesia, both of which are likely to be in the grain length range for allele C of *GS3*, differ in grain length and do so reliably. Furthermore, the basmati rices around 8.0 mm and KDML105 at 6.8 mm both carry the A-allele of *GS3* [46]. Another gene associated with grain length, *GL3*, has recently been cloned [48], but its presence has not been associated widely with grain length in diverse germplasm. We therefore have four reliable phenotypes of grain length (Figure 2A), and two genotypes at the *GS3* loci, but as other QTLs and genes such as *GL3* are further understood, the complete genetic regulation of grain length should become known.

Grain shape is often used to describe the physical dimensions of grains. This is the ratio of length and width, and three classes are defined: bold (<2), medium (2.1 – 3), and slender (>3). Confusingly, these terms could also describe width. Figure 2B shows the percentage of each grain shape category for each length class for all the varieties in the present paper. Interestingly, as grain length increases, the proportion of slender grains increases and the proportion of bold grains decreases. However, Fitzgerald et al. (2009) showed a grain with length and width 11 and 3.8 mm respectively, which places an extra-long grain into the medium shape classification. This suggests that selection has driven the trend seen in Figure 2B, but the diversity of rice could encompass all grain shapes for all length classes. If the rice community intends to continue using grain shape to describe the dimensions of the grain, it would be useful to also always include the length in order to avoid confusion.

### Amylose content

Amylose, a linear polymer of glucose units linked primarily by α-1,4 linkages, influences texture and the potential of cooked grains to retrograde after cooking. This is also one of the major traits used in the selection process for eating quality among rice breeding programs [41]. Hence, most of the INQR members were familiar with the amylose classes popular in their region.

**Figure 3:**
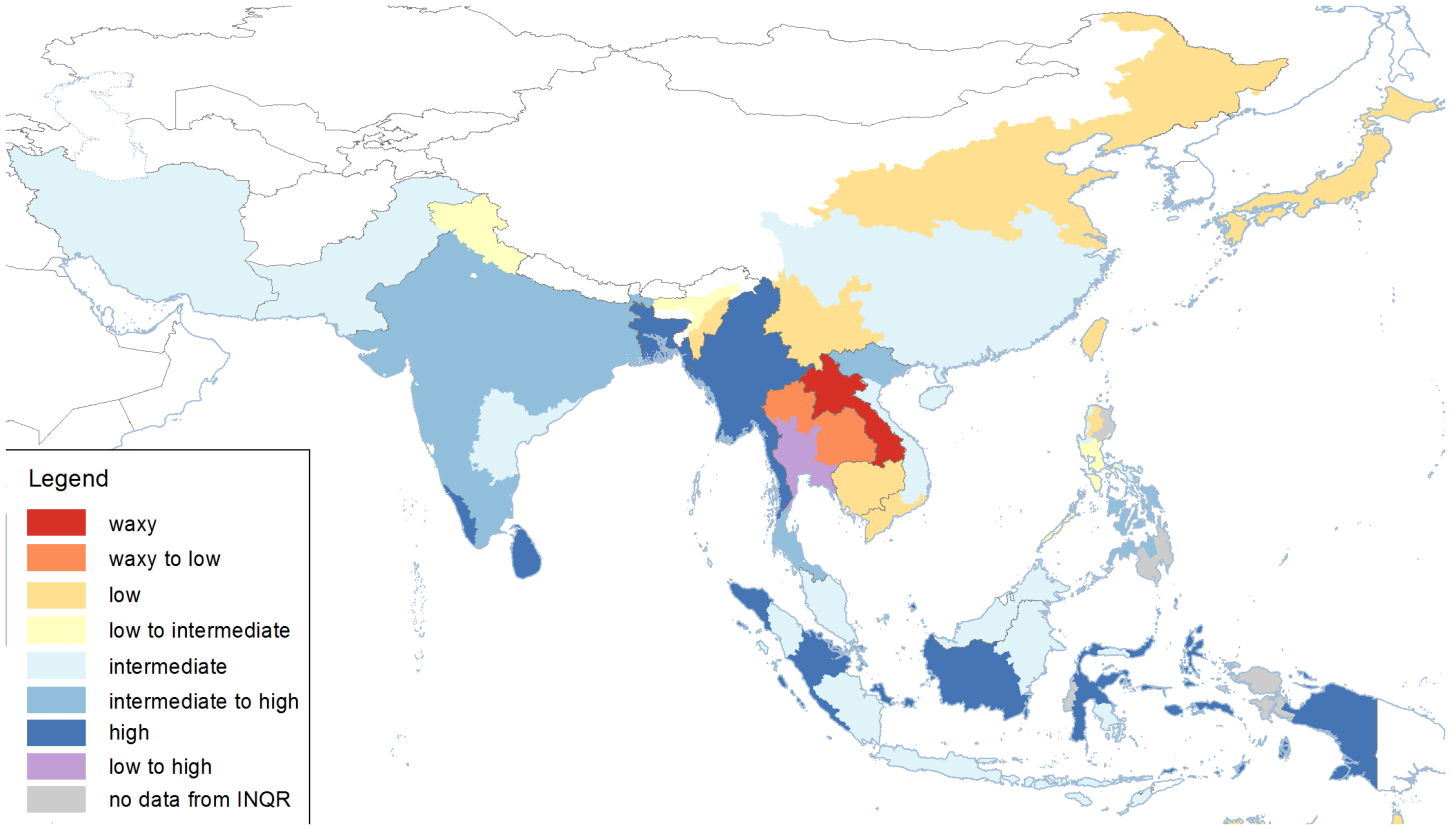
Regional variation in amylose content of the three most popular varieties in the countries, states, and provinces of Asia. In some regions, two types of amylose class are preferred. Additional information for other regions can be found in Table 2. Data were obtained from INQR representatives from each region.

Consumers in Lao PDR and the Isan region of Thailand prefer waxy or sticky rice (Figure 3). In Japan, Taiwan, Cambodia, Thailand, parts of Lao PDR, Egypt and Australia, consumers prefer low amylose rice, as do consumers in the northern and south-western provinces of China, and southern Vietnam (Figure 3, Table 1). Rice with intermediate amylose content is preferred in Iran, Pakistan, Malaysia, Philippines, many states in India, and some provinces of China, Vietnam, Indonesia and Uruguay. High amylose varieties are popular in Myanmar, Sri Lanka, provinces of Indonesia, and many states of India (Figure 3). High AC rice is also preferred in Ghana, Senegal, Suriname, Colombia and parts of Uruguay (Table 1).

**Table 1:** Primers used to sequence *Wx* gene of low AAC varieties.

Primer Name	Sequence (5'-3')	Size (bp)
Wx_ex1F	CAATGCAACGTACGCCAAG	19
Wx_ex1R	CCTGGGTGTGTTTCTCTAGACTC	23
Wx_ex2aF	GTGGGCTAGCTGACCTAGATTTG	23
Wx_ex2aR	TGTTTAAGGTTTGGTGAGCCTA	22
Wx_ex2bF	CCAAGAAACTGCTCCTTAAGTCC	23
Wx_ex2bR	GTACCTGTCTGCAACCTTGATCT	23
Wx_ex3-6F	GCATTGGATGGATGTGTAATGT	22
Wx_ex3-6R	GGCTGGTAGTTGTTCTTCAGGT	22
Wx_ex6-9F	GGAAGCATCACGAGTTTACCAT	22
Wx_ex6-9R	CTTGCCTTGTCAGAATCAATCA	22
Wx_ex10-11F	CAACCACGGTAAGAACGAATG	21
Wx_ex10-11R	AGGGCTGGAGAAATCAACAAG	21
Wx_ex12-13F	CTGCAGGGGATGAGATACG	19
Wx_ex12-13R	TGTAGATCTCAGGCTCTTCAAGG	23
Wx_ex14F	TGTTTGCAACATGGATTTCAGGG	23
Wx_ex14R	TCCTGAGTCAAACTACTGCTCCT	23

**Table 2:** Grain quality traits in non-Asian rice-growing countries.

Country	Length	Shape	Amylose	Aroma	Gel Con	Gel Temp
Australia	Medium and long,	Medium	Low	1 of 3		Low and intermediate
Egypt	Medium and long,	Slender and medium	Low			
Ghana	Medium and long,	Slender	High	1 of 3	Soft and intermediate	Low and intermediate
Uganda	Medium and long	Slender and medium	Intermediate and high	1 of 3	Soft and intermediate	Intermediate to high
Senegal	Medium and long,	Slender and medium	High		Soft	Intermediate and high
Portugal	Long and medium	Slender and medium	intermediate			Intermediate and high
Suriname	Extra-long,	Slender	High			Low
Chile	Short and long,	Bold	Intermediate			Low
Colombia	Long	Slender	High			Low and intermediate
Brazil	Long		Intermediate and high		Intermediate	Intermediate and high
Uruguay	Long	Slender	Intermediate and high			Low and intermediate
USA	Medium and long	Slender and medium	Low and intermediate			Low and intermediate

Some countries do not measure all traits.

The amylose classes are associated with polymorphisms in the *Waxy* gene (*Wx*) [49] which encodes for the granule-bound starch synthase (GBSSI) enzyme that is responsible for amylose synthesis [50]. A G-T polymorphism at the 5’ splice site at intron 1 results in two functional alleles *Wx^a^* and *Wx^b^*, which differentiate low amylose from high and intermediate classes [51]. An A-C polymorphism in exon 6, *Wx^in^* discriminates intermediate from high amylose [52], [53]. The waxy phenotype is a null mutation with a 23-bp duplication in exon 2 which causes a frame-shift resulting in non-functional GBSSI protein [54] and no amylose [41], [55].

Each class of amylose is a range of 4 – 12 percentage points. Figure 3 has been plotted on the basis of the traditionally-defined classes. However, data from different countries indicated that popular rice varieties span each range. For example, the low amylose varieties from Thailand and Cambodia are 12–15% amylose, whereas the low amylose varieties from Japan, China, Korea and Australia are about 18–19% amylose. These varieties are all the same Waxy haplotype, with the single-nucleotide polymorphism (SNP) at intron 1. The same variation within class was found for the intermediate and high amylose classes.

**Figure 4:**
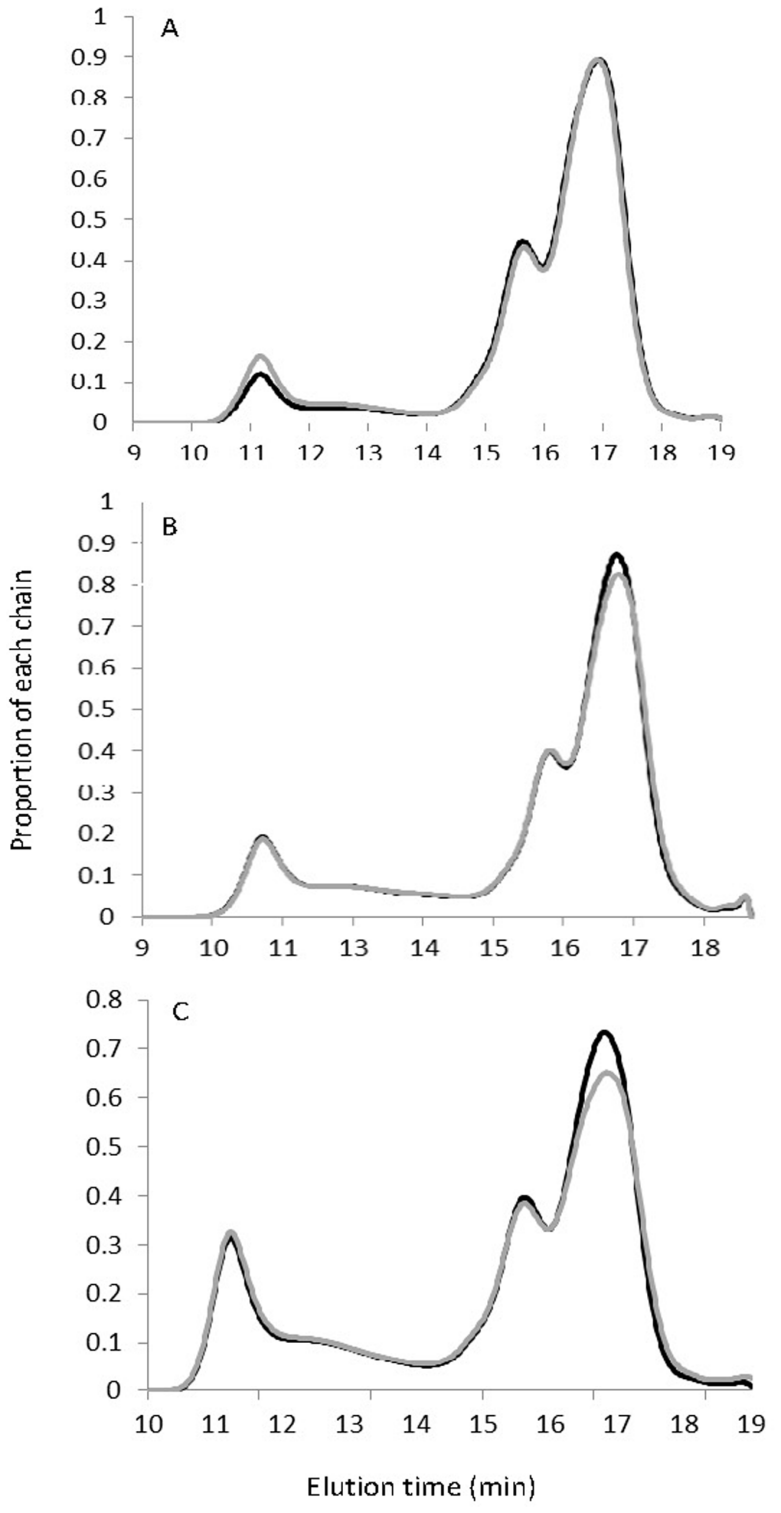
Size exclusion chromatograms of low (A), intermediate (B) and high (C) amylose varieties. Each curve is the average of ten different rice varieties from each end of the range in each class. In each class, those with high amylose are shown by the grey curves, and those with lower amylose are shown by the black curve. Chains of amylose elute before 14 min and amylopectin chains after 14 min.

In order to visualise the amylose content more clearly, varieties with amylose content across the range of each class were analysed using size exclusion chromatography (SEC). The SEC traces clearly demonstrate that within a typical amylose class, different levels of amylose and amylopectin were observed (Figure 4). For the low amylose class there was a clear group of lower amylose varieties, which were the Thai and Cambodian *indica* varieties, and a clear group with higher amylose which are the Australian and Japanese *temperate japonica* varieties. However, the range in the other two classes is due to a difference in the amount of amylopectin chains (Figure 4). Those at the upper end of the intermediate and high amylose class had a significantly lower percentage of amylopectin than those at the lower end of the amylose class (Figure 4). Since only the low amylose haplotype showed differences in the amylose chains, the coding region of the *Wx* gene was sequenced for each, to determine the presence of another allele. However, no sequence differences were found, suggesting either that the difference in amylose is due to loci other than *Wx*, interactions relating to amylose that are specific to either the *indica* or *tropical japonica* germplasm class, or that the amylose content is lower when this haplotype is grown under tropical conditions. This allele of the Wx gene leading to low amylose is sensitive to high temperature [56]. However, it is possible that main climate difference between the countries could be humidity, although no relationship between amylose content and humidity has yet been demonstrated. For intermediate and high amylose, the data indicate that amylopectin synthesis is also involved in amylose content, and presents an opportunity to discover the biochemical and genetic bases of varying amylopectin content.

The range in amylose content of varieties within each class suggests that an additional sub-categorisation within each amylose class would be valuable to define clearly the differences within rice that belong to the same amylose haplotype. Low amylose rice could be further classified into subclass A and B with amylose content 11–15% and 16–19%, respectively. Similarly, the intermediate amylose group may be divided into subclass A with amylose contents between 20–22%, and subclass B with 23–24% amylose. High amylose can also be categorised into subclass A and B with amylose content 25–27% and >27%, respectively. Although the underlying genetics for each sub-class has not been elucidated, it would be valuable to adopt these descriptors to facilitate understanding in the global rice literature.

### Gel consistency

Gel consistency is a test that was developed to differentiate between the texture of cooked rice from the high and intermediate amylose classes [34]. It measures the distance travelled by a gel after cooking and was developed for varieties of 24–30% amylose [34]. Gel consistency is not measured in a number of countries because high amylose rices are not part of the rice improvement program.

**Figure 5:**
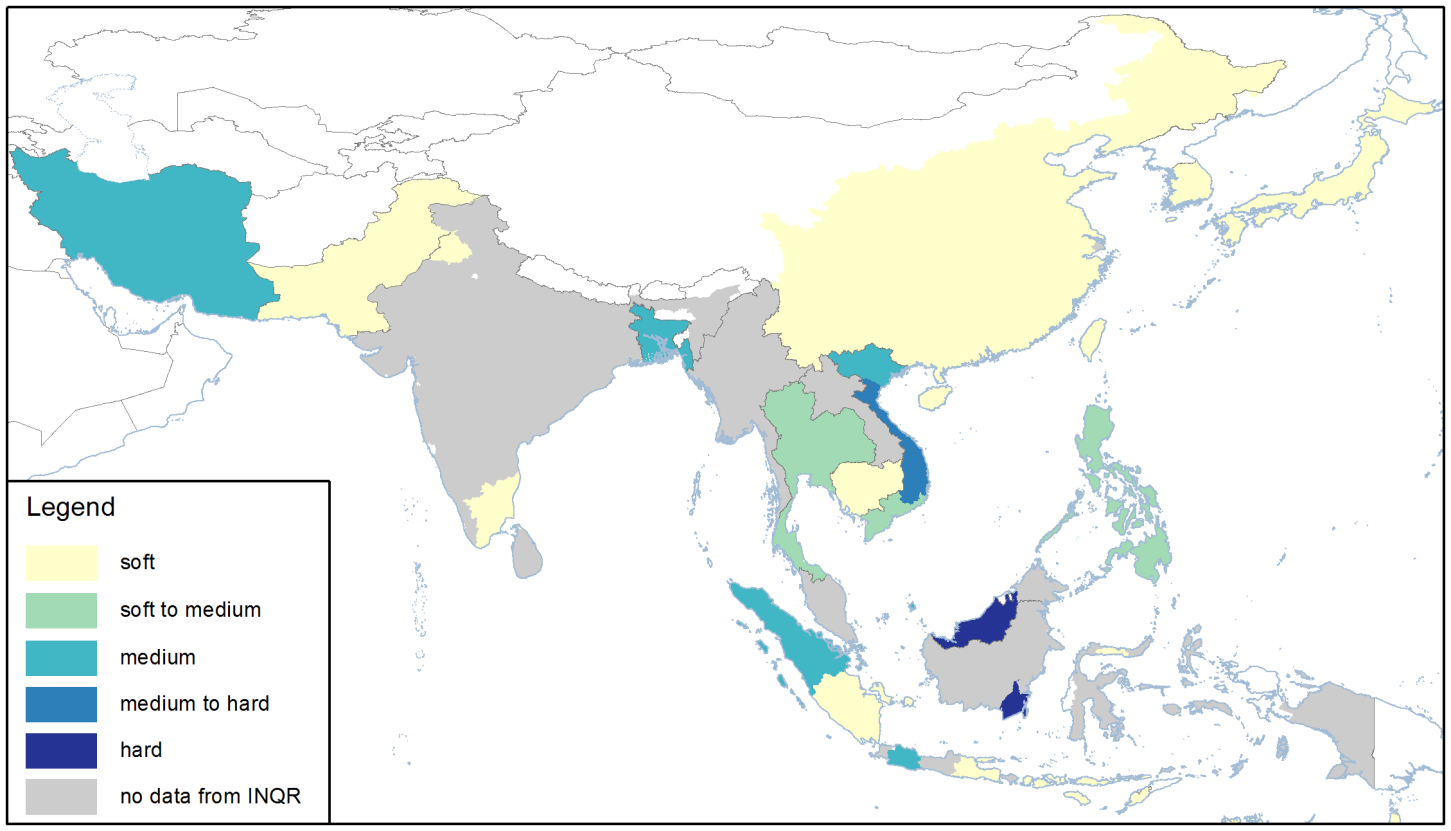
Consumer preferences for texture based on gel consistency values. In many countries and regions, gel consistency is not measured (grey). Additional information for the other regions can be found in Table 2. Data obtained from INQR representatives from each region.

It was expected that only the rice improvement programs developing high and intermediate amylose would be using a gel consistency test, such as Iran, Pakistan, Thailand, Philippines, Tamil Nadu in India, Sarawak in Malaysia and Kalimantan in Indonesia and some others (Figure 5). However other countries also reported values for gel consistency (Figure 5, Table 1), even those where low amylose varieties are developed. Most countries that reported gel consistency values prefer soft or intermediate gel consistency, which indicates soft-texture in varieties with high amylose. The genetic basis of gel consistency is a SNP in exon 10 of the Waxy gene [42]. This SNP has only been associated with gel consistency for varieties with a functional intron 1 [42], which excludes the lower amylose varieties.

### Gelatinisation temperature

**Figure 6:**
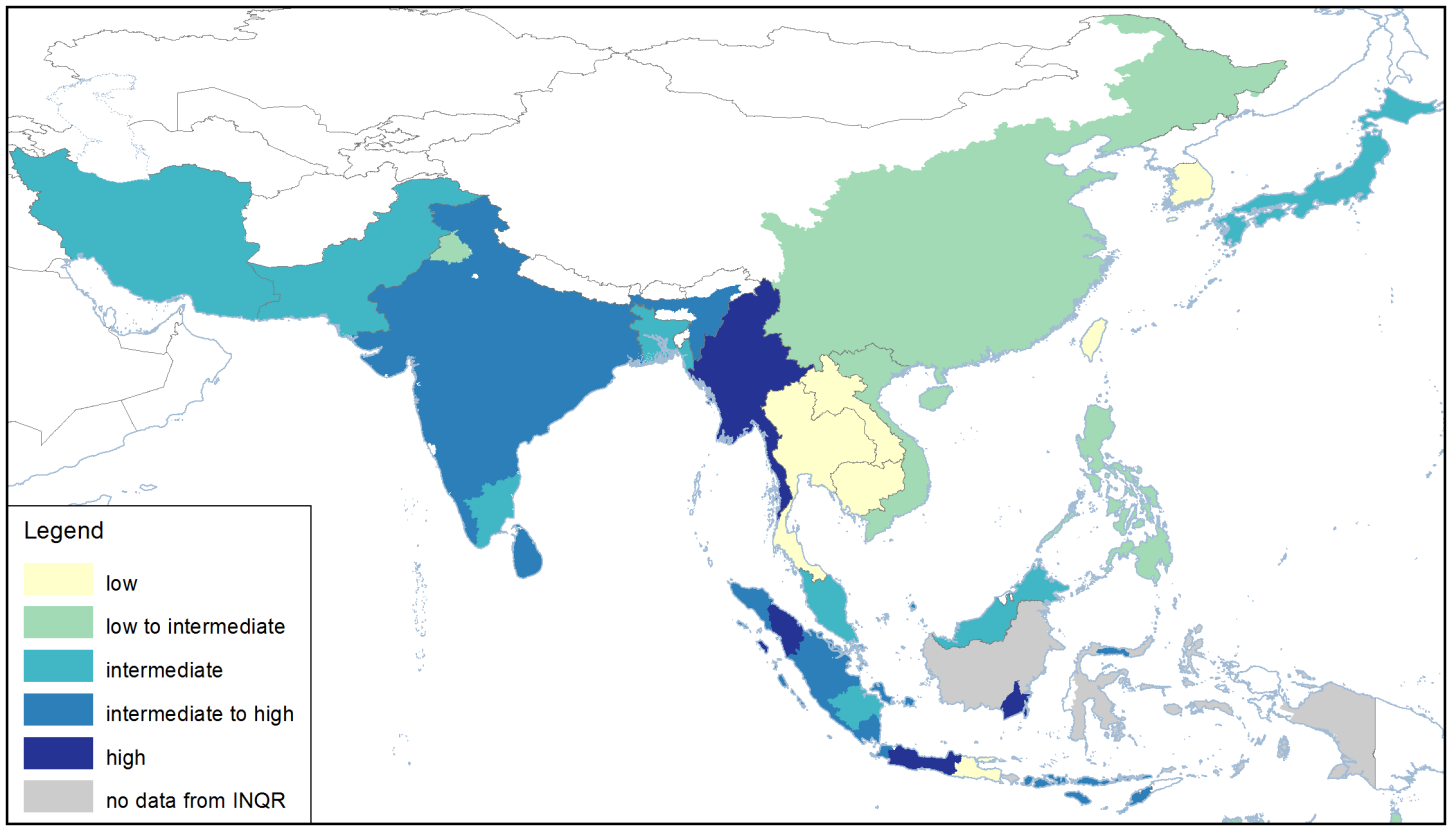
Regional variation in gelatinisation temperature of the three most popular varieties in the countries, states, and provinces of Asia. In some regions, two classes are preferred. Additional information for other regions can be found in Table 2. Data were obtained from INQR representatives from each region.

Gelatinisation temperature describes the temperature that starch granules begin to melt, and the grains begin to cook [57]. It ranges from 55 – 85°C in domesticated rice, and correlates with the cooking time of rice and the final cooked texture [32]. In the countries of South and Central Asia, gelatinisation temperature is intermediate to high, which means the rice takes about 4 min longer to cook than rice with low gelatinisation temperature, such as is found in some Southeast Asian countries (Figure 6).

Gelatinisation temperature is divided into two groups by allelic variation in *SSlla* [58]–[60]. The SNPs in *SSlla* define four haplotypes [58], [61] and two haplotypes associate with high and two with low gelatinisation temperature [32]. Intermediate gelatinisation temperature varieties are found in all haplotype groups [32], suggesting that another locus interacts with *SSlla* to produce the intermediate phenotype.

Many of the popular varieties have been genotyped for the *SSlla* locus [32], and the genotype associates with the phenotypes reported in Figure 6, in all cases.

The additional cooking time required for rice of high gelatinisation temperature leads to an enormous amount of extra energy used when expressed on a population basis. It has been estimated that each minute less cooking time globally represents 2500 years cooking time saved per day [12]. Thus if all countries could lower the gelatinisation time, and thereby cooking time of their popular varieties, this would lead to significant savings of fuel, and a measureable reduction in the carbon footprint of rice.

### Aroma

Aromatic rice varieties are of great interest in the market because they command a higher price than non-aromatic rice. The two types of aromatic rice are basmati and jasmine. Aromatic rice is characterised by the presence of a popcorn / baked bread-like flavour compound called 2-acetyl-1-pyrroline (2AP). Although generally only present in very low amounts, its low odour threshold means that 2AP is easily detected by consumers [62].

In both jasmine and basmati rice breeding programs, the presence of 2AP is used to select fragrant progeny. 2AP is determined subjectively either by sniffing a gram of rice grains that have been soaked for an hour in potassium hydroxide, quantitatively by gas chromatography, or by detecting the mutation in the gene that associates with aroma [35].

**Figure 7:**
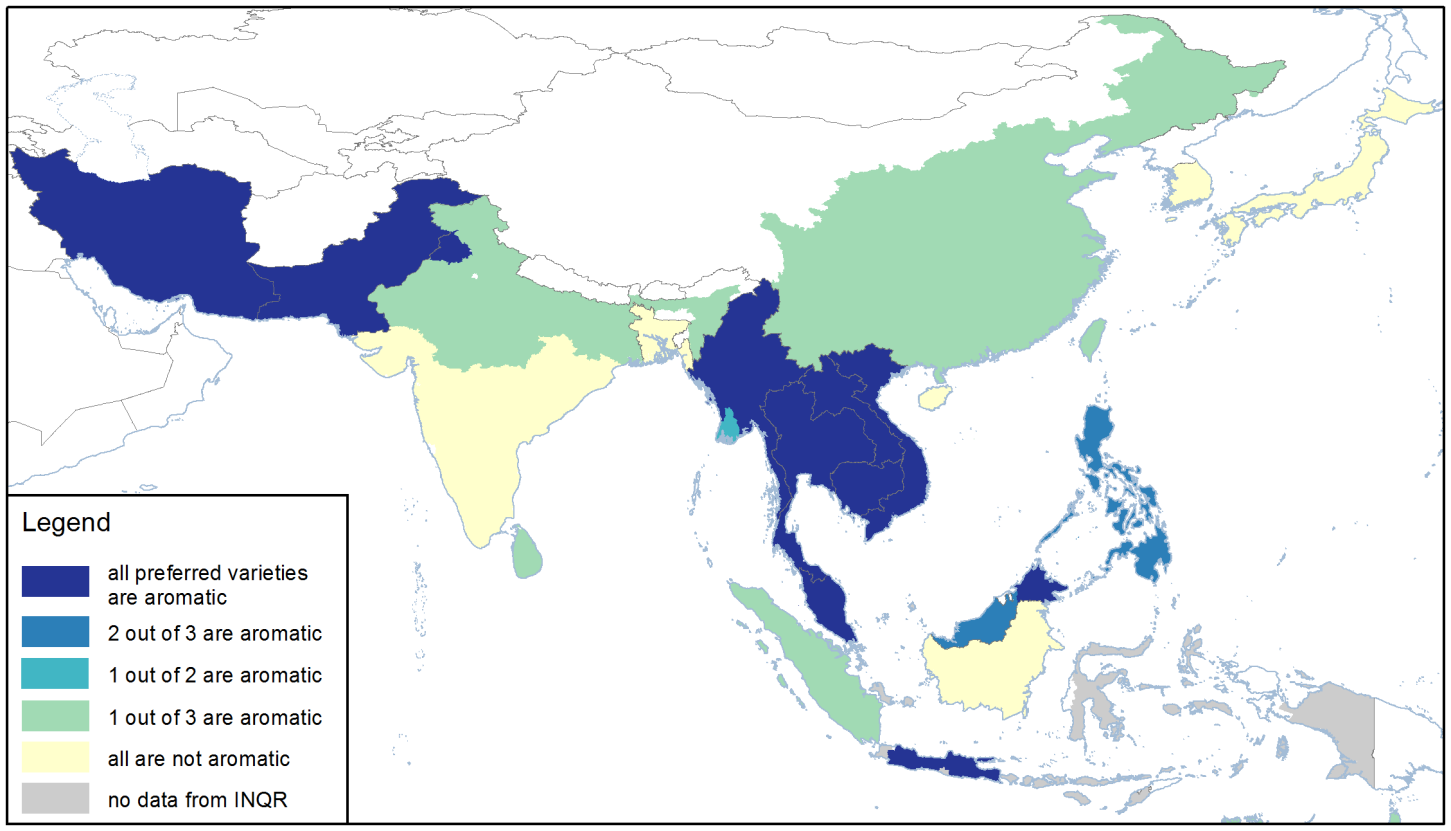
Consumer preferences for aromatic rice for the countries, states, and provinces of Asia. In some regions, all popular rices are aromatic, in others, one or two is aromatic, and in some countries, aromatic rice is not popular. Additional information for other regions can be found in Table 2. Data were obtained from INQR representatives from each region.

Figure 7 shows that aromatic rice is very important in the Greater Mekong Subregion (GMS), Malaysia, Iran, Pakistan, the states of Punjab and Haryana of India and some provinces of Indonesia. In Iran, the aromatic varieties are sadri types, which group together genetically with the basmati rices of Pakistan and India [63]. Most of those varieties are traditional, and the improved varieties grown in Pakistan and India are derived from traditional basmati parents. The fragrant rices in the GMS are *indica* jasmine types, and these are mostly traditional varieties. The taste and aroma of South and Central Asian and the GMS jasmine rices are distinctly different, although all have 2AP and all have been shown to carry the common mutation in the fragrance gene [35].

**Figure 8:**
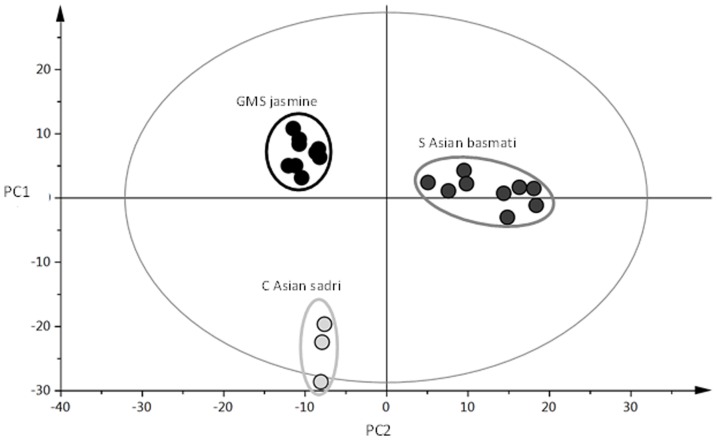
Principal Components Analysis of the volatile metabolomic signature of the traditional indica varieties from the Greater Mekong Subregion (GMS), basmati varieties from South Asia, and the sadri varieties from Iran. PC1 explains 27% and PC2 explains 21% of variation.

The fragrance of rice is a function of the volatile compounds emitted from the grains. Deep analysis of a selection of the sadri rices from Iran, the basmati rices from Pakistan, Punjab and the jasmine rices from the GMS shows that there are distinct differences in the volatile profile between the three germplasm classes (Figure 8). Moreover, in a sensory analysis previously carried out using the same popular varieties discussed here, the sadri rices were characterised as sweet dairy, the Thai *indica* rices as popcorn and the basmati as haylike [64], consistent with the different volatile signatures observed in Figure 8.

Rice fragrance is almost always defined based on the presence of 2AP. However, Figure 8, showing the metabolomic signatures of the varieties in this paper, and other studies working with jasmine and basmati types [65], [66] have shown that other compounds and fragrance descriptors also contribute to the aroma of jasmine and basmati rices. Given the importance of these three classes of rice to Iran, Pakistan and to some states of India, and the countries in the GMS, it seems that improving yields in those regions requires a deeper understanding of the important compounds of fragrance for each type, and research to deliver robust phenotyping tools that are able to distinguish between the three types of aroma.

### Grain quality combinations

**Figure 9:**
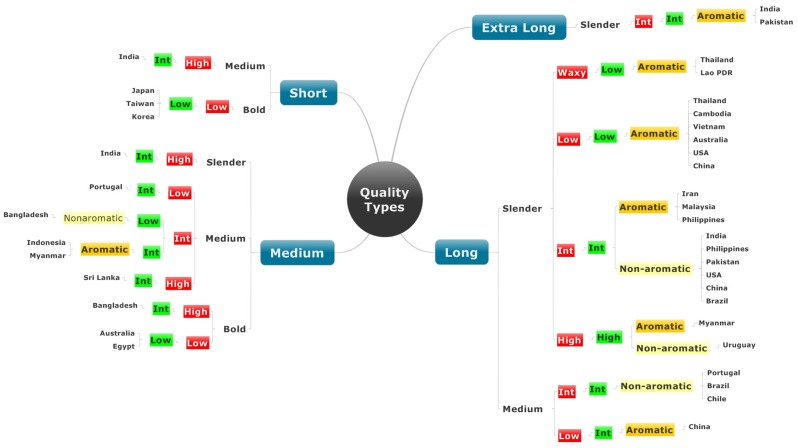
Number of different quality types in each grain length class based on combinations of the current tools for measuring quality: grain length (blue), shape, amylose content (red), gelatinisation temperature (green), and the presence of aroma.

Using the information for the quality traits we currently measure, the number of different combinations of quality were determined (Figure 9). Immediately, the complexity of local preference becomes evident. Based on the combinations, there were 18 grain types found, spanning Asia, Australia, and the main rice producing countries of Europe, Africa and the Americas. For short grain, there are just two types of quality; for medium length grain, there are seven types, with one type being aromatic, represented by Pandan Wangi from Indonesia and Paw San Hmwe from Myanmar. For the extra long grains, there is just one type, and these are the basmati rices from Pakistan and the north-western states of India. The highest number of combinations was found in the long grain class (Figure 9), and five of the eight combinations are aromatic.

Figure 9 shows that consumer preferences from several countries are described by exactly the same combinations of grain quality. Some of these make sense, such as the long, slender, waxy, aromatic varieties from Isan in Thailand and Lao PDR since these two regions spent many years together in the ancient Kingdom of Siam. However, other common combinations cannot be explained by common heritage. For example, BRS Primavera from Brazil has exactly the same combinations of quality as IR64 from the Philippines (Figure 9). However, a previous study undertaken by the INQR included these two varieties and found that their taste, flavour and texture differ significantly [64]. There are many such examples in Figure 9 that can be compared in the same way. This indicates that our suite of phenotyping tools for grain quality is insufficient to distinguish fully the properties that consumers use to make decisions.

## Conclusion

By mapping the different quality traits for each country, the INQR has shown that there are at least 18 different quality types of rice that are favoured around the world. The two most popular combinations, that are found all over the world, are both long and slender, while one type has low amylose, low gelatinisation temperature and is aromatic, and the other has intermediate amylose and gelatinisation temperature and is not aromatic. Our previous study showed that the same combinations of measured quality traits are still different when eaten by consumers [64]. This strongly indicates that quality evaluation teams are not able to provide sufficient information to enable breeders to select for market quality, and suggests that new science needs to be brought to rice quality to develop new trait combinations of aroma, flavour and texture that match market demands. This paper presents a framework for classifying the global variation in rice grain quality, which can be further enumerated as required, and reinforces the need for improved objective measurement within each quality category to capitalise on the considerable investment in variety development and to meet better the needs of a growing population of discerning consumers demanding high quality rice.

## Supporting Information

Figure S1 **Spatial units depicting level of detail of data on preferred rice traits.**
(TIF)

Table S1 **Varieties nominated by each country as most popular.**
(DOCX)

Table S2 **Rice consumption per capita per country from 2004 – 2009 (FAOStat 2013).**
(DOCX)
