# Supplementary figures and images for: Diversity of Global Rice Markets and the Science Required for Consumer-Targeted Rice Breeding
Source: PLoS One. 2014 Jan 14;9(1):e85106. doi: 10.1371/journal.pone.0085106 (PMC3893639; doi:10.1371/journal.pone.0085106)

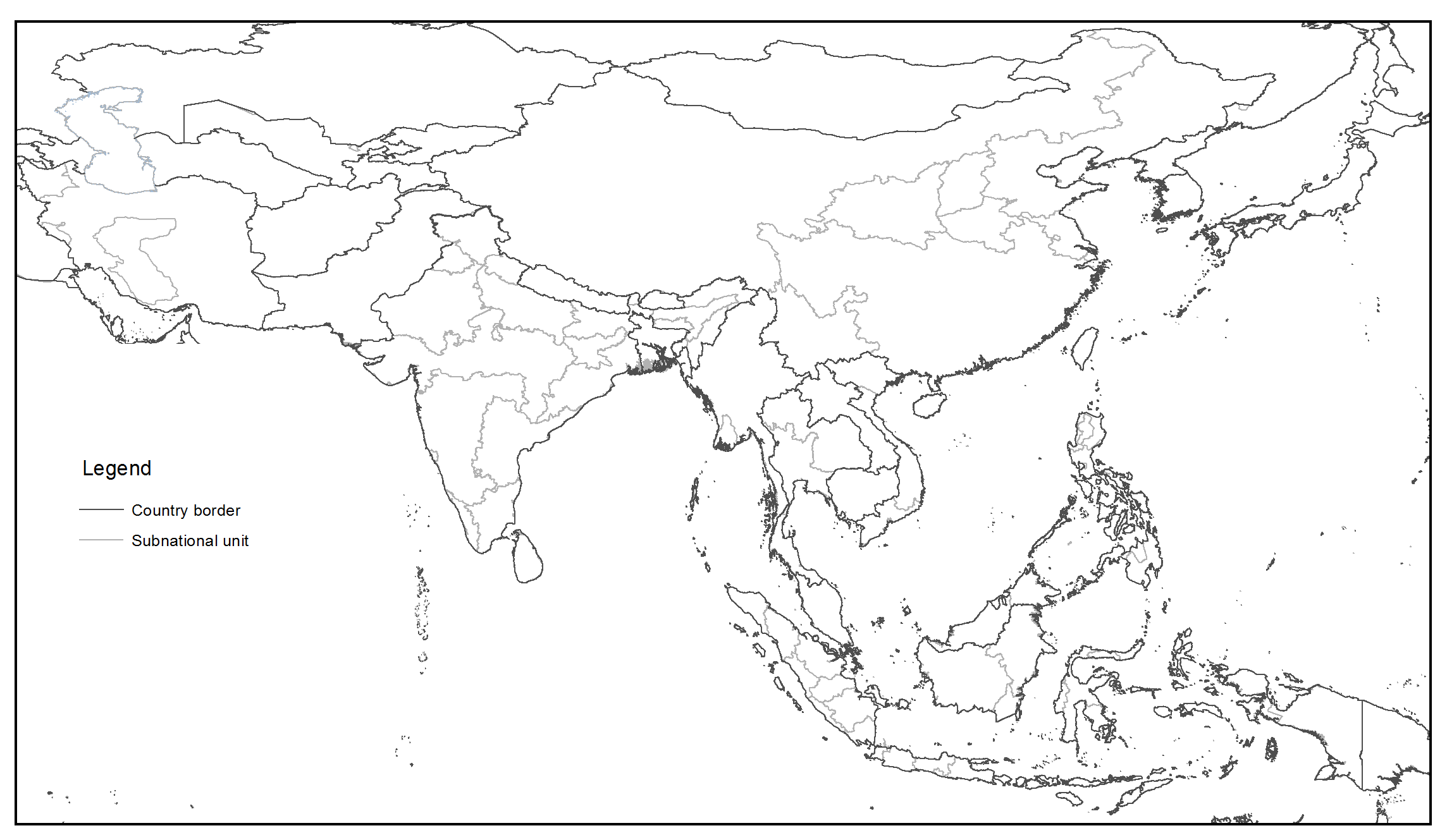

Supplement: Figure S1 — Spatial units depicting level of detail of data on preferred rice traits. (TIF) [file pone.0085106.s001.tif]
