# Supplementary material for: Diversity of Global Rice Markets and the Science Required for Consumer-Targeted Rice Breeding
Source: PLoS One. 2014 Jan 14;9(1):e85106. doi: 10.1371/journal.pone.0085106 (PMC3893639; doi:10.1371/journal.pone.0085106)
Supplement: Table S1 — Varieties nominated by each country as most popular. (DOCX) [file pone.0085106.s002.docx]

| **Country** | **Nominated varieties** | **Country** | **Nominated varieties** |
| --- | --- | --- | --- |
| Punjab (North India) | Pusa Basmati 1121 | VietNam | Jasmine 85 |
|  | Basmati 386 |  | OM4900 |
|  | Punjab Basmati 2 |  | OM7347 |
| Central (India) | Taroari basmati | Iran | Hashemi |
|  | Pusa 1121 |  | Shirodi |
|  | BPT 5204 |  | Tarom |
|  | Kalanamak |  | Fajv |
| Tamil Nadu (India) | ADT 43 | Cambodia | Phka malis |
|  | ADT45 |  | Phka romduol |
|  | Improved White Ponni |  | Phka kngnei |
| Andra Pradesh (India) | Sambha masuri | Egypt | Sakha 105 |
|  | Swarna |  | Sakha 106 |
| Pakistan | Super basmati |  | Sakha 107 |
|  | Basmati 515 | Ghana | Wakatsuki |
|  | Basmati 2000 |  | Sikamo |
| China | Zhongzheyou1 |  | Nerica 1 |
|  | Zhongjian 2 | Uganda | Jinja |
|  | Xiangwanxian13 |  | Supa |
|  | Ningjing43 |  | Pearl-kenya-pishori |
| Bangladesh | BR11 |  | Kaisho |
|  | BRRI Dhan28 |  | Nerica |
|  | BRRI Dhan29 | Senegal East | Sahel177 |
| Indonesia | Ciherang |  | Sahel328 |
|  | Pandanwangi |  | Nerica-S-19 |
|  | Rojolele |  | Nerica-S-21 |
| Japan | Koshihikari |  | Nerica-S-36 |
|  | Hitomebore |  | ITA-150 |
|  | Hinohikari |  | WAB_56-50 |
| Laos | KDML 105 | Australia | Amaroo |
|  | Homesavan |  | Langi |
| Malaysia | MRQ 74 |  | Reiziq |
|  | MR 219 | Suriname | ADRON-125 |
|  | MR 220 |  | ADRON-128 |
| Malaysia - Sarawak | Bario |  | ADRON-130 |
|  | Bajong | Portugal | Ariete |
|  | Biris |  | Albatross |
| Myanmar | Paw San Hmwe | Uruguay | INIA Tacuarí |
|  | Manawthukha |  | INIA Olimar |
|  | Aye Yar Min |  | El Paso 144 |
| Philippines | Sinandomeng | Chile | Diamante INIA |
|  | Dinorado |  | Zafiro INIA |
|  | IR64 |  | Oro |
| Sri Lanka | Bg 300 | Colombia | Oryzica 1 |
|  | Bg 352 |  | Fedearroz50 |
|  | Bg 358 |  | Fedearroz60 |
| Taiwan | Koshihikari | Brazil | BRS Primavera |
|  | Taikeng 9 |  | Epagri 108 |
|  | Tainung 71 |  | IRGA417 |
| Thailand | KDML105 | USA | Wells |
|  | PTT1 |  | CL 151 |
|  | RD6 |  | Jupiter |

Table S1: Varieties nominated by each country as most popular.
