# Supplementary material for: Diversity of Global Rice Markets and the Science Required for Consumer-Targeted Rice Breeding
Source: PLoS One. 2014 Jan 14;9(1):e85106. doi: 10.1371/journal.pone.0085106 (PMC3893639; doi:10.1371/journal.pone.0085106)
Supplement: Table S2 — Rice consumption per capita per country from 2004 – 2009 (FAOStat 2013). (DOCX) [file pone.0085106.s003.docx]

Table S2: Rice consumption per capita per country from 2004 – 2009 (FAOStat 2013).
